# Technical Success and Mid-Term Outcomes of Endovascular Revascularization of Tibio-Peroneal Trunk Lesions

**DOI:** 10.3390/jcm10163610

**Published:** 2021-08-16

**Authors:** Sanne W. de Boer, Patricia A. H. van Mierlo-van den Broek, Jean-Paul P. M. de Vries, Simone F. Kleiss, Gijs C. Bloemsma, Debbie A. B. de Vries-Werson, Bram Fioole, Reinoud P. H. Bokkers

**Affiliations:** 1 Department of Radiology, Maastricht University Medical Center, 6229 HX Maastricht, The Netherlands; 2 CARIM School for Cardiovascular Diseases, Maastricht University, 6229 HX Maastricht, The Netherlands; 3 Department of Vascular Surgery, Maasstad Hospital, 3079 DZ Rotterdam, The Netherlands; BroekP2@maasstadziekenhuis.nl (P.A.H.v.M.-v.d.B.); fiooleb@maasstadziekenhuis.nl (B.F.); 4 Department of Surgery, Division of Vascular Surgery, University of Groningen, University Medical Center Groningen, 9700 RB Groningen, The Netherlands; j.p.p.m.de.vries@umcg.nl (J.-P.P.M.d.V.); s.f.kleiss@umcg.nl (S.F.K.); d.a.b.de.vries-werson@umcg.nl (D.A.B.d.V.-W.); 5 Department of Radiology, Medical Imaging Center, University of Groningen, University Medical Center Groningen, 9700 RB Groningen, The Netherlands; g.c.bloemsma@umcg.nl (G.C.B.); r.p.h.bokkers@umcg.nl (R.P.H.B.)

**Keywords:** endovascular therapy, infrapopliteal, tibio-peroneal trunk, chronic limb threatening ischemia

## Abstract

Tibio-peroneal trunk (TPT) lesions are usually categorized as ‘complex’ in anatomical classifications, which leads to the perception that endovascular therapy (EVT) will be challenging and the outcome most likely poor. This multicenter, retrospective cohort study investigates the efficacy of the EVT of TPT lesions in patients with chronic limb threatening ischemia (CLTI) or an infrapopliteal bypass at risk. The primary endpoint was limb-salvage. The secondary outcomes were technical success, freedom from clinically driven target lesion revascularization (CD-TLR), overall survival, and amputation-free survival. A total of 107 TPT lesions were treated in 101 patients. At 3 years, the limb-salvage rate was 76.4% (95% CI 66.0–86.8%). Technical success was achieved in 96.3% of cases. The freedom from CD-TLR, amputation-free survival, and overall survival at 3 years were 53.0% (95% CI 38.1–67.9%), 33.6% (95% CI 23.0–44.2%), and 47.7% (95% CI 36.1–59.3%), respectively. Reintervention significantly increased the hazard ratio for amputation by 7.65 (95% CI 2.50–23.44, *p* < 0.001). Our results show that the EVT of both isolated and complex TPT lesions is associated with high technical success and acceptable limb-salvage rates, with reintervention being a major risk factor for amputation. Moreover, mid-term mortality rate was relatively high. In future revisions of the anatomical grading scales, the classification of TPT lesions as highly complex should be reconsidered.

## 1. Introduction

Peripheral artery occlusive disease (PAOD) is common and often disabling. Over the last decades, the prevalence of PAOD has increased by 23.5%, largely due to global aging [1]. The first clinical manifestation is intermittent claudication, which in 15% of patients gradually progresses into chronic limb-threatening ischemia (CLTI) [1]. Patients with advanced CLTI are at high risk of major amputation and have a reported mortality rate of 25% within the first year after diagnosis [2,3].

Revascularization therapy is the primary treatment for patients with CTLI. For patients with steno-occlusive lesions in the popliteal and infrapopliteal arteries, endovascular therapy (EVT) has become the preferred treatment, and is associated with similar limb-salvage rates, reduced morbidity, higher cost-effectiveness, and shorter hospitalization as compared to surgery [2,3,4,5,6].

The involvement of the tibio-peroneal trunk (TPT) is frequently observed in extensive multi-vessel disease. Lesions in the TPT are considered anatomically complex and are therefore regarded as being more suited for surgical revascularization or conservative treatment with the best medical management, as stated in the anatomical classifications used in consensus guidelines [2,7]. However, given the developments within EVT over the last decade, femoro-tibial lesions are more frequently revascularized with endovascular means.

The aim of this study is to investigate the efficacy of the EVT of TPT lesions in patients with CLTI or an infrapopliteal bypass at risk in terms of technical success, the need for target lesion revascularization, and amputation-free survival.

## 2. Materials and Methods

This is a multicenter retrospective cohort study of patients receiving EVT for TPT lesions at three centers: two university teaching hospitals (Maastricht University Medical Center and University Medical Center Groningen) and one community teaching hospital (Maasstad Hospital, Rotterdam).

The study protocol was reviewed by the medical ethics committees of all participating hospitals. Due to the retrospective nature of the study, the need to obtain individual informed consent according to the Dutch Medical Research Involving Human Subjects Act was waived. This study was conducted in accordance with good clinical practice and the applicable national and European laws.

### 2.1. Patient Population

Patients who received the EVT of the TPT for infrapopliteal CTLI (defined as Rutherford IV–VI), or for the treatment of an infrapopliteal bypass at risk, were eligible for inclusion. Exclusion criteria were acute limb ischemia, dissection, a popliteal aneurysm with associated occlusion of the TPT, or the absence of clinical, imaging, and procedural data.

Demographic information, clinical details, and follow-up data were obtained from electronic patient files. A multidisciplinary team of vascular surgeons and interventional radiologists assessed patients according to standard clinical care as defined by the Dutch Society for Surgery and Interventional Radiology [8]. Preprocedural imaging was performed with duplex ultrasound (DUS), computed tomography angiography (CTA), or magnetic resonance angiography (MRA).

### 2.2. Procedure Techniques

Endovascular treatment was performed in an angiographic suite or hybrid operating room under local anesthesia or, if deemed necessary, under conscious sedation or general anesthesia. The procedures were performed using either an antegrade percutaneous femoral arterial access or a crossover approach with a contralateral retrograde femoral access, followed by a diagnostic lower limb digital subtraction angiography (DSA) to plan the treatment. A primary distal retrograde approach of the lesion via either the dorsal pedal artery, the anterior tibial artery or the posterior tibial artery was left at the operator’s discretion. A retrograde approach was always combined with antegrade access. Proximal inflow lesions in the iliac or femoro-popliteal arteries were always treated first when present.

TPT lesions were primarily treated with percutaneous transluminal angioplasty (PTA). Stent placement was used as a bail-out option in case of flow limiting dissections, >30% residual stenosis, or elastic recoil after PTA. The use of specific guidewires, support catheters, and angioplasty balloons were chosen at the operator’s discretion. For TPT lesions, either a 0.018-inch or a 0.014-inch platform was used. In general, PTA balloons were sized 1:1 to the non-diseased arterial diameter of the segment proximal or distal of the lesion, and the duration of balloon inflation time was 120 s.

All treated lesions were scored according to the Global Limb Anatomic Staging System (GLASS) classification by 3 experienced interventionalists (SB, BF, and RB) [2]. Calcium burden was scored in an anteroposterior angiographic view into three categories: no visible calcium, medium (unilateral calcified), or heavy (bilateral calcifications). This was derived from the peripheral arterial calcium scoring system (PACSS) for the femoro-popliteal arteries [9]. The pedal inflow was graded on a post-procedural angiogram and could be scored as: (1) inline flow to the foot, (2) indirect vascularization, defined as revascularization of another angiosome in the presence of collaterals to the affected angiosome, or (3) no pedal inflow. 

Procedural details were retrieved from the electronic patient files and radiology information and picture archiving and communication systems by means of a standardized case report form.

### 2.3. Follow-Up

After the endovascular intervention, all patients were clinically assessed at regular intervals in a vascular outpatient clinic. Patients with ulcers were evaluated 2 weeks after EVT. Patients with ulcers were also referred to a physiatrist to evaluate the need for offloading. In case no ulcers were present, a patient was evaluated after 6 weeks. The assessment consisted of a physical examination, the evaluation of wound healing, and the measurements of the ankle-brachial index and toe pressures. Additional imaging with DUS, CTA, or MRA was performed in cases of insufficient wound healing or persisting clinical symptoms due to ischemia. All patients received clopidogrel as monotherapy, unless otherwise indicated.

### 2.4. Outcome Measures

The primary outcome measure was limb-salvage, defined as the freedom from major lower extremity amputation (above-ankle amputation). Secondary outcome measures included technical success defined as a successful lesion treatment with less than 30% residual stenosis [10], freedom from clinically driven target lesion revascularization (CD-TLR), amputation-free survival (AFS), which is a composite endpoint that combines the outcomes of the endpoints mortality and major amputation, and all-cause mortality. All procedural and periprocedural complications occurring up to 30 days post-intervention were registered.

### 2.5. Statistical Analysis

Continuous data are presented as median ± interquartile range; categorical data are provided as counts (percentage). Kaplan–Meier (KM) estimates are used to obtain survival estimates for freedom from CD-TLR, limb-salvage, amputation-free survival and freedom from all-cause mortality. Survival estimates are given with a 95% confidence interval (CI). In patients who had the event of interest, the follow-up ended at the date of diagnosis of the event. In patients who did not have an event, observations were censored at the last follow-up visit. All KM estimates are truncated when the numbers at risk are less than 10. Hazard ratio analyses for limb-salvage and freedom from CD-TLR are performed using univariable and multivariable Cox proportional hazards analyses or Cox proportional hazards with a time-dependent covariate. All data were analyzed using SPSS software (version 25 for Windows; IBM Corporation, Armonk, NY, USA).

## 3. Results

One hundred and one consecutive patients with 107 target lesions in the TPT were included in the study. Five patients received treatment in both legs at different points in time, and one patient received treatment in both limbs on the same day. Patient characteristics and comorbidities are listed in Table 1. Of all patients, 79.2% were classified as Rutherford 5 or 6. In 15.1% of patients, the Rutherford class was 4, and in 5.7% of patients, the Rutherford class was 3. All patients with Rutherford class 3 had an infrapopliteal bypass at risk.

The TPT lesion characteristics are listed in Table 2. Of the 107 lesions, 39.3% were isolated to the TPT, 27.1% consisted of a TPT lesion continuous into a single tibial artery, and 33.6% of the lesions involved the bifurcation or all tibial arteries. For the infrapopliteal segment, lesions were classified according to GLASS as: class 1, 30.8%; class 2, 12.1%; class 3, 7.5%; and class 4, 49.5%. An occlusion was present in 22.4% of the lesions, and heavy, bilateral calcifications were present in 38.3% of the lesions.

The median follow-up in this cohort was 10.6 months (interquartile range (IQR) 4.2–24.0), with 92 (86.0%) patients having completed ≥12-month follow-up and 82 (76.6%) patients having completed ≥24-month follow-up. A total of 48 (45.8%) patients died during follow-up.

### 3.1. Endovascular Therapy

The procedural outcomes are listed in Table 3. A technical success of 96.3% was reported. Of the four patients without technical success, all were treated by conservative means; eventually, two patients underwent a major amputation after 4 months and 7 years, respectively. A plain old balloon angioplasty (POBA) was performed in 96.3% of the procedures, and drug-coated balloons (DCB) were used in 3.7% of procedures. Bail-out stenting was required in 6.5% of the procedures, with a bare-metal stent (BMS) used in six procedures and a drug-eluting stent (DES) in one procedure. In 72.9% of the procedures (*n* = 78), an antegrade approach of the lesion was chosen. In the remaining 26.2% of procedures (*n* = 28), an antegrade approach was combined with retrograde access.

Inline flow to the foot was reported in 35.5%, which is mainly explained by the high rate of limited or absent preprocedural vascularization of the foot. Indirect vascularization was reported in 53.3%, and pedal inflow was reported in 9.3%.

### 3.2. Efficacy and Safety Outcomes

**Figure 1:**
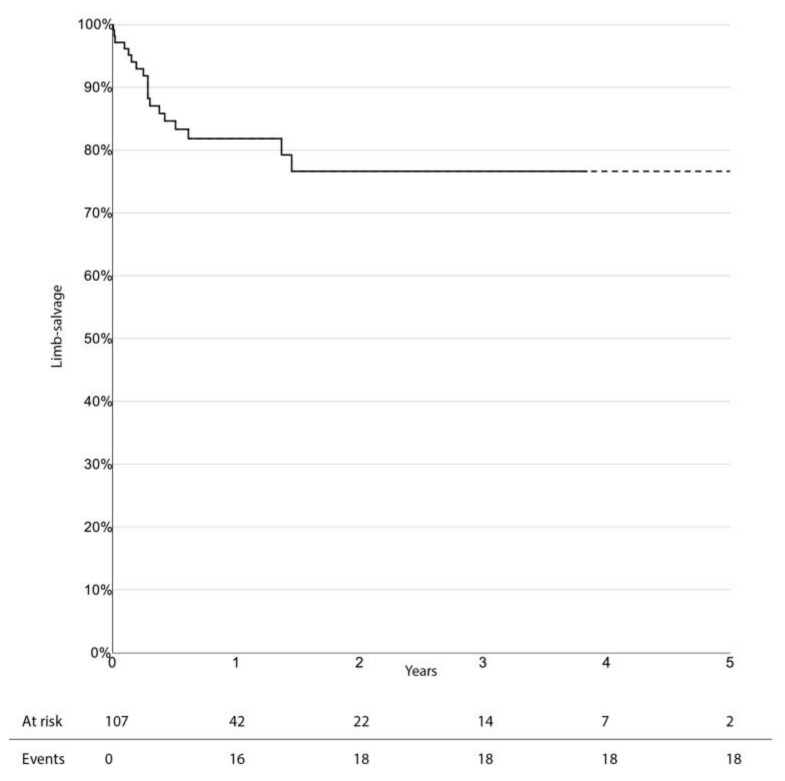
Survival estimate for limb-salvage rates.

**Figure 2:**
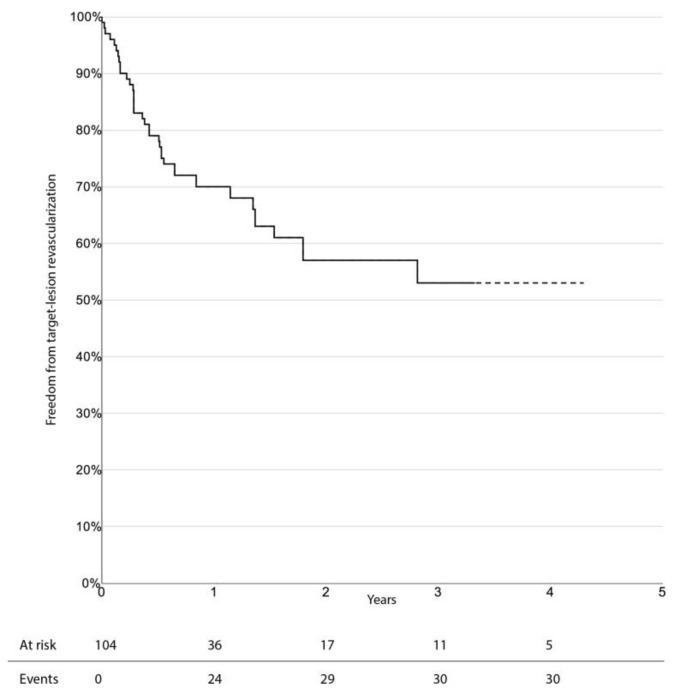
Survival estimate for freedom from target-lesion revascularization.

**Figure 3:**
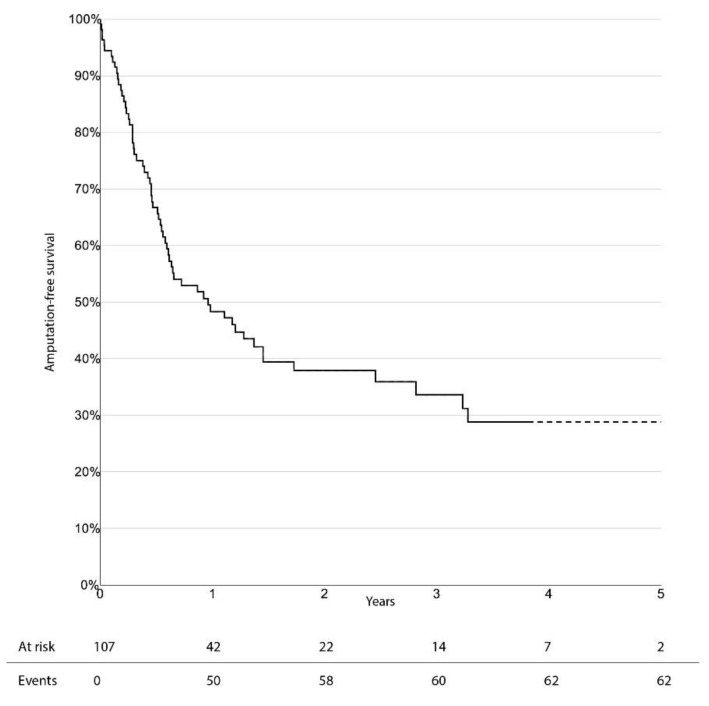
Survival estimate for amputation-free survival.

**Figure 4:**
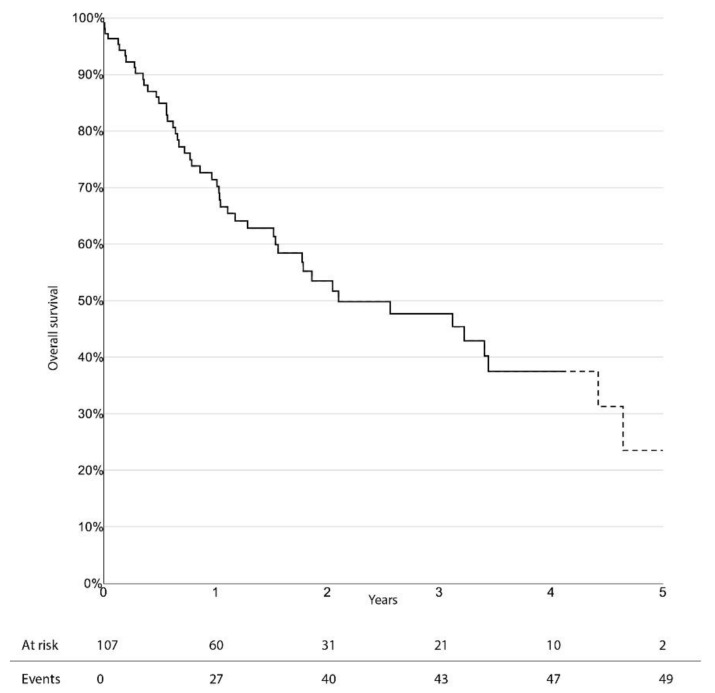
Survival estimate for overall survival.

The limb-salvage rate at 3 years was 76.4% (95% CI 66.0–86.8%). Freedom from CD-TLR, amputation-free survival, and overall survival at 3 years were 53.0% (95% CI 38.1–67.9%), 33.6% (95% CI 23.0–44.2%), and 47.7% (95% CI 36.1–59.3%), respectively. There was no significant difference in survival estimates between diabetic and non-diabetic patients, regarding limb-salvage rate and freedom from CD-TLR. Kaplan–Meier curves for limb-salvage rate, freedom from CD-TLR, amputation-free survival, and overall survival are shown in Figure 1, Figure 2, Figure 3 and Figure 4, respectively.

The univariable hazard ratios for all analyzed risk factors for amputation and reintervention are listed in Table 4. Reintervention as a time-dependent covariate significantly increased the hazard ratio (HR) for amputation (HR amputation 7.65, 95% CI 2.50–23.44, *p* < 0.001). A TPT lesion continuing into a single artery showed a non-significant increase in the HR for both major amputation and reintervention (HR amputation 2.46, 95% CI 0.69–8.71, *p* = 0.165; HR reintervention 1.85, 95% CI 0.78–4.37, *p* = 0.160). The continuation of the TPT lesion into either the bifurcation or the involvement of all tibial arteries showed a non-significant increase in the HR for amputation to 2.47 (95% CI 0.74–8.23; *p* = 0.140). Diabetes led to an increased univariable hazard ratio of 1.741 (95% CI 0.827–3.663; *p* = 0.144) for reintervention and an increased univariable hazard ratio of 1.687 (95% CI 0.653–4.356; *p* = 0.280) for major amputation. A multivariable model could not be fitted due to the small number of events.

## 4. Discussion

This study evaluated the clinical outcomes of the EVT of patients suffering CLTI or a bypass at risk with a lesion in the TPT. Even though a high number of lesions could be classified as complex according to the GLASS classification in a fragile patient population, a high rate of technical success and limb-salvage was achieved.

Limb preservation is an important factor in the quality of life of CLTI patients. Besides the best medical treatment, and a multidisciplinary team approach [11,12], revascularization is often needed to avoid a major amputation. Limb-salvage rates after infrapopliteal endovascular recanalization are reported between 65% and 98.4% after 1 year [13,14], with technical success rates varying from 70 to 100% [14,15,16,17,18]. These outcomes are not directly comparable to the results of the current study, because they pertain almost exclusively to tibial lesions as opposed to isolated or continuous TPT lesions. Considering that TPT lesions are frequently graded and perceived as high-complex lesions, the current study shows that the efficacy of the endovascular recanalization of isolated and continuous TPT lesions is similar to that found in other studies, with a high technical success rate and 3-year limb-salvage rates. The amputation-free 3-year survival rate of 33.6% may be explained mainly by a mortality rate of 52.3%. Most patients included in this study are high-risk patients with substantial comorbidities. The high mortality rate is comparable to that found in a large cohort of CLTI patients from the Vascular Quality Initiative [19]. In this cohort, high-risk patients showed a 3-year mortality rate of 48%.

The TPT can be seen as a continuation of the peroneal and posterior tibial artery, but bifurcation and trifurcation lesions can be more complex, as they may require the recanalization of at least two arteries. This may have led to high complexity classifications in anatomical grading scales like GLASS, which can be debated. In 2018, a large retrospective cohort study was published, and the results suggested a distinction between big artery disease (BAD), small artery disease (SAD), and foot artery disease (FAD) [20]. The study indicated that FAD and SAD could play a pivotal role in developing tissue ulcers and that these differences should be considered in any revascularization strategy. Since TPT lesions are a big artery disease, it seems logical that the efficacy outcomes and technical success are comparable to other infrapopliteal “big artery” studies reporting on tibial segments. In the current study, infrapopliteal GLASS 4, most often due to a CTO of the TPT, was not associated with an increased risk of amputation or reintervention.

Traditionally, due to the minimal invasive character of EVT, reinterventions are considered benign and easily accessible [21,22]. The current study, however, showed that a patient receiving a reintervention is at a very high risk of eventually being amputated with a HR of 7.65. This is in line with a study published by Utsunomiya et al. in 2018, in which similar results were seen in patients with CLTI on dialysis, with a reported HR of 3.35 [23]. After an endovascular revascularization, the median wound healing time is reported to be 97 days [24]. When restenosis occurs, this will inhibit blood flow and tissue perfusion, delaying this process. Even with a close monitoring of the wound, there will be a delay between the onset of restenosis and reintervention. This delay increases the time that a limb is subjected to an inadequate blood flow and tissue perfusion, increasing the chances of infection and an eventual amputation.

This raises two questions; firstly, whether endovascular specialists need to be more eager during the primary treatment session in order to prevent a reintervention; and secondly, since almost all amputations were within the first year after the index procedure, do infrapopliteal target-lesions after EVT need to be monitored more closely by targeted DUS? Regarding the first question, in the current study POBA was performed in 96.3% of the lesions with a high technical success rate, and only a few instances of bail-out stenting were necessary. Recoil follows PTA over a 15 to 30 min period [25], but in general a control DSA is performed within seconds to minutes after PTA. Waiting for a longer period of time helps identify elastic recoil and whether additional PTA or stent placement is necessary. Regarding the post-treatment monitoring, this is traditionally done by clinical evaluation, and ankle-brachial index and toe-pressure measurements. Although these are adequate markers for the failure of EVT, up to one-third of clinically significant failures are missed without a targeted DUS [26], and an abnormal DUS within the first 30 days after an infrainguinal intervention is associated with an increased risk of amputation [27].

## 5. Limitations

The retrospective character of this study is a limitation. Although consecutive cases were included in this study, it may be likely that patients were treated conservatively during this period, potentially leading to inclusion bias. In addition, not all follow-up data were standardized or available, and could not be retrieved for all patients. For this reason, it was not possible to include patient reported outcome measures like quality of life scores and pain scores, or wound, ischemia, and foot infection scores and wound healing. Moreover, not all procedural data have been recorded, and for this reason it was not possible to provide a detailed overview of the exact materials used or the median balloon inflation times.

Calcium burden was scored using a pragmatic approach, derived from the PACSS classification (no visible calcium, (medium) unilateral calcifications, or (heavy) bilateral calcifications). This is a non-standardized, non-validated method of visual scoring and may lead to observer bias.

Finally, even though this study included 107 lesions in 101 patients, the small sample size is a limiting factor in the identification of significant risk factors for limb-survival or reintervention.

## 6. Conclusions

This retrospective multicenter study shows that the EVT of both isolated and complex TPT lesions is associated with high technical success and limb-salvage rates, with reintervention being a major risk factor for amputation. In future revisions of the anatomical grading scales, the classification of TPT lesions as highly complex should be reconsidered.

## Figures and Tables

**Table 1:** Patient characteristics.

Variable		No. (*n* = 106)
Age, y		77.0 [68–83]
Male sex		64 [60.4%]
Rutherford classification		
	Category 3	6 [5.7%]
	Category 4	16 [15.1%]
	Category 5-6	84 [79.2%]
Risk factors		
	Diabetes	54 [50.9%]
	Smoking, current or recent	23 [21.7%]
	Hypertension	72 [67.9%]
	Hypercholesterolemia	27 [25.5%]
	Chronic pulmonary disease	22 [20.8%]
	Coronary artery disease	62 [58.5%]
	Congestive heart failure	46 [43.4%]
	Hemodialysis	10 [9.4%]
	Past stroke	16 [15.1%]
	Body mass index (kg/m^2^)	25.4 [23.1–28.6]

Age and body mass index are provided as median [IQR]. Other variables are provided as counts [%].

**Table 2:** Lesion characteristics.

Variable		No. (*n* = 107)
Occlusion		24 [22.4%]
Anatomical location		
	Isolated TPT	42 [39.3%]
	TPT lesion continuing into single artery	29 [27.1%]
	TPT lesion continuing into bifurcation	24 [22.4%]
	TPT lesion continuing into ATA, PTA and PA	12 [11.2%]
TPT lesion calcifications		
	No calcifications	30 [28.0%]
	Medium, unilateral	31 [29.0%]
	Heavy, bilateral	41 [38.3%]
	Not reported	5 [4.7%]
Preprocedural crural outflow arteries		
	0	20 [18.7%]
	1	54 [50.5%]
	2	26 [24.3%]
	3	7 [6.5%]
GLASS Femoro-popliteal		
	0	40 [37.4%]
	1	16 [15.0%]
	2	13 [12.1%]
	3	12 [11.2%]
	4	26 [24.3%]
GLASS infrapopliteal		
	1	33 [30.8%]
	2	13 [12.1%]
	3	8 [7.5%]
	4	53 [49.5%]
GLASS inframalleolar		
	P0	29 [27.1%]
	P1	58 [54.2%]
	P2	14 [13.1%]
	Not reported	6 [5.6%]
GLASS Total score		
	I	22 [20.6%]
	II	24 [22.4%]
	III	61 [57.0%]

Provided as counts [%]; TPT, tibio-peroneal trunk; ATA, anterior tibial artery; PTA, posterior tibial artery; PA, peroneal artery; GLASS, Global Limb Anatomic Staging System.

**Table 3:** Procedural characteristics.

Variable		No. (*n* = 107)
TPT lesion approach		
	Antegrade	78 [72.9%]
	Antegrade combined with retrograde	28 [26.2%]
	Not reported	1 [0.9%]
Devices used		
	POBA	103 [96.3%]
	Drug-coated balloon	4 [3.7%]
	Bail-out bare-metal stent	6 [5.6%]
	Bail-out drug-eluting stent	1 0.9%]
Technical success		103 [96.3%]
Post-procedural pedal inflow		
	Inline flow to the foot	38 [35.5%]
	Indirect vascularization via collaterals *	57 [53.3%]
	No pedal inflow	10 [9.3%]
	Not reported	2 [1.9%]

TPT, tibio-peroneal trunk; POBA, plain old balloon angioplasty; * Defined as revascularization of another angiosome in the presence of collaterals to the affected angiosome.

**Table 4:** Unadjusted hazard ratios for major amputation and reintervention.

Variable	Major Amputation	*p*	Reintervention	*p*
	HR [95% CI]		HR [95% CI]	
TPT occlusion	0.59 [0.14–2.56]	0.449	0.85 [0.32–2.27]	0.741
Isolated TPT lesion (reference)				
TPT lesion + single artery	2.46 [0.69–8.71]	0.165	1.85 [0.78–4.37]	0.160
TPT lesion + bifurcation or all 3 arteries	2.47 [0.74–8.23]	0.140	1.09 [0.45–2.62]	0.854
No calcifications (reference)				
Medium, unilateral calcifications	1.55 [0.44–5.48]	0.500	1.12 [0.36–3.48]	0.850
Heavy, bilateral calcifications	1.46 [0.44–4.92]	0.527	1.87 [0.73–4.78]	0.194
Number of outflow vessels preprocedural	1.19 [0.68–2.05]	0.545	1.16 [0.76–1.76]	0.504
GLASS infrapopliteal (category 1 = reference)				
Category 2	0.41 [0.05–3.40]	0.408	0.51 [0.11–2.35]	0.389
Category 3	1.50 [0.30–7.46]	0.618	2.68 [0.91–7.89]	0.073
Category 4	0.90 [0.32–2.52]	0.836	0.83 [0.36–1.89]	0.651
GLASS inframalleolar (P0 = reference)				
P1	1.19 [0.41–3.45]	0.743	1.42 [0.59–3.39]	0.437
P2	0.79 [0.15–4.10]	0.783	1.67 [0.49–5.72]	0.416
Inline flow to the foot (reference category)				
Indirect vascularization	0.90 [0.34–2.37]	0.836	0.91 [0.42–1.98]	0.808
No pedal outflow	0.66 [0.08–5.40]	0.702	2.34 [0.73–7.46]	0.151
Reintervention *	7.65 [2.50–23.44]	<0.001	-	-

HR, hazard ratio; CI, confidence interval; TPT, tibio-peroneal trunk; GLASS, Global Limb Anatomic Staging System; * analyzed as time-dependant covariate.

## Data Availability

The data presented in this study are available on request from the corresponding author. The data are not publicly available due to privacy and ethical reasons.

## References

[1] Fowkes F.G.R., Rudan D., Rudan I., Aboyans V., Denenberg J.O., McDermott M.M., Norman P.E., Sampson U.K., Williams L.J., Mensah G.A. (2013). Comparison of global estimates of prevalence and risk factors for peripheral artery disease in 2000 and 2010: A systematic review and analysis. Lancet.

[2] Conte M.S., Bradbury A.W., Kolh P., White J.V., Dick F., Fitridge R., Mills J.L., Ricco J.-B., Suresh K.R., Murad M.H. (2019). Global vascular guidelines on the management of chronic limb-threatening ischemia. J. Vasc. Surg..

[3] Conte S.M., Vale P.R. (2018). Peripheral Arterial Disease. Heart Lung Circ..

[4] Fanelli F., Cannavale A., Gazzetti M., Lucatelli P., Wlderk A., Cirelli C., D’Adamo A., Salvatori F.M. (2014). Calcium burden assessment and impact on drug-eluting balloons in peripheral arterial disease. Cardiovasc. Interv. Radiol..

[5] Aboyans V., Ricco J.-B., Bartelink M.-L.E.L., Björck M., Brodmann M., Cohnert T., Naylor A.R., Roffi M., Tendera M., Vlachopoulos C. (2018). Editor’s Choice—2017 ESC Guidelines on the Diagnosis and Treatment of Peripheral Arterial Diseases, in collaboration with the European Society for Vascular Surgery (ESVS). Eur. J. Vasc. Endovasc. Surg..

[6] Romiti M., Albers M., Brochado-Neto F.C., Durazzo A.E.S., Pereira C.A.D.B., De Luccia N. (2008). Meta-analysis of infrapopliteal angioplasty for chronic critical limb ischemia. J. Vasc. Surg..

[7] Norgren L., Hiatt W.R., Dormandy J.A., Nehler M.R., Harris K.A., Fowkes F.G.R. (2007). Inter-Society Consensus for the Management of Peripheral Arterial Disease (TASC II). J. Vasc. Surg..

[8] Vahl A.C., Teijink J.A.W., Elsman B.H.P., Reekers J.A., Monajemi H., Bartelink M.E.L., Auwerda A., Rouwet E., Stokvis H. Perifeer Arterieel Vaatlijden (PAV) 2016. https://richtlijnendatabase.nl/richtlijn/perifeer_arterieel_vaatlijden_pav/pav_-_startpagina.html.

[9] Rocha-Singh K.J., Zeller T., Jaff M.R. (2014). Peripheral arterial calcification: Prevalence, mechanism, detection, and clinical implications. Catheter. Cardiovasc. Interv..

[10] Diehm N., Pattynama P., Jaff M., Cremonesi A., Becker G., Hopkins L., Mahler F., Talen A., Cardella J., Ramee S. (2008). Clinical Endpoints in Peripheral Endovascular Revascularization Trials: A Case for Standardized Definitions. Eur. J. Vasc. Endovasc. Surg..

[11] Gabel J., Bianchi C., Possagnoli I., Mehta R., Abou-Zamzam A.M., Teruya T., Kiang S., Bishop V., Valenzuela A. (2020). Multidisciplinary approach achieves limb salvage without revascularization in patients with mild to moderate ischemia and tissue loss. J. Vasc. Surg..

[12] Steunenberg S.L., De Vries J., Raats J.W., Thijsse W.J., Verbogt N., Lodder P., Van Eijck G.-J., Veen E.J., De Groot H.G., Ho G.H. (2018). Quality of Life and Mortality after Endovascular, Surgical, or Conservative Treatment of Elderly Patients Suffering from Critical Limb Ischemia. Ann. Vasc. Surg..

[13] Iida O., Soga Y., Hirano K., Kawasaki D., Suzuki K., Miyashita Y., Terashi H., Uematsu M. (2012). Long-term results of direct and indirect endovascular revascularization based on the angiosome concept in patients with critical limb ischemia presenting with isolated below-the-knee lesions. J. Vasc. Surg..

[14] Mustapha J., Finton S., Diaz-Sandoval L. (2016). Percutaneous Transluminal Angioplasty in Patients with Infrapopliteal Arterial Disease Systematic Review and Meta-Analysis. Circ. Cardiovasc. Interv..

[15] van Overhagen H., Spiliopoulos S., Tsetis D. (2013). Below-the-knee interventions. Cardiovasc. Radiol..

[16] Mustapha J.A., Anose B.M., Martinsen B.J., Pliagas G., Ricotta J., Boyes C.W., Lee M.S., Saab F., Adams G. (2020). Lower extremity revascularization via endovascular and surgical approaches: A systematic review with emphasis on combined inflow and outflow revascularization. SAGE Open Med..

[17] Haider S.N., Kavanagh E.G., Forlee M., Colgan M.P., Madhavan P., Moore D.J., Shanik G.D. (2006). Two-year outcome with preferential use of infrainguinal angioplasty for critical ischemia. J. Vasc. Surg..

[18] Biagioni R., Biagioni L.C., Nasser F., Burihan M.C., Ingrund J.C., Neser A., Miranda F. (2018). Infrapopliteal Angioplasty of One or More than One Artery for Critical Limb Ischaemia: A Randomised Clinical Trial. Eur. J. Vasc. Endovasc. Surg..

[19] Simons J.P., Schanzer A., Flahive J.M., Osborne N.H., Mills J.L., Bradbury A.W., Conte M.S. (2019). Survival prediction in patients with chronic limb-threatening ischemia who undergo infrainguinal revascularization. J. Vasc. Surg..

[20] Ferraresi R., Mauri G., Losurdo F., Troisi N., Brancaccio D., Caravaggi C.M., Neri L. (2018). BAD transmission and SAD distribution: A new scenario for critical limb ischemia. J. Cardiovasc. Surg..

[21] Ryer E.J., Trocciola S.M., Derubertis B., Lam R., Hynecek R.L., Karwowski J., Bush H.L., Mureebe L., McKinsey J.F., Morrissey N.J. (2006). Analysis of outcomes following failed endovascular treatment of chronic limb ischemia. Ann. Vasc. Surg..

[22] Lumsden A.B., Davies M.G., Peden E.K. (2009). Medical and endovascular management of critical limb ischemia. J. Endovasc. Ther..

[23] Utsunomiya M., Iida O., Yamauchi Y., Nakano M., Soga Y., Kawasaki D., Takahara M., Nakamura M. (2016). Influence of Repeat Intervention on the Risk of Major Amputation After Infrapopliteal Angioplasty for Critical Limb Ischemia. J. Endovasc. Ther..

[24] Iida O., Nakamura M., Yamauchi Y., Kawasaki D., Yokoi Y., Yokoi H., Soga Y., Zen K., Hirano K., Suematsu N. (2013). Endovascular treatment for infrainguinal vessels in patients with critical limb ischemia: OLIVE registry, a prospective, multicenter study in Japan with 12-month follow-up. Circ. Cardiovasc. Interv..

[25] Baumann F., Fust J., Engelberger R.P., Hügel U., Do D.-D., Willenberg T.A., Baumgartner I., Diehm N. (2014). Early recoil after balloon angioplasty of tibial artery obstructions in patients with critical limb ischemia. J. Endovasc. Ther..

[26] Saarinen E., Laukontaus S., Albäck A., Venermo M. (2014). Duplex surveillance after endovascular revascularisation for critical limb ischaemia. Eur. J. Vasc. Endovasc. Surg..

[27] Humphries M.D., Pevec W.C., Laird J.R., Yeo K.K., Hedayati N., Dawson D.L. (2011). Early duplex scanning after infrainguinal endovascular therapy. J. Vasc. Surg..

